# Immune state is associated with natural dietary variation in wild mice *Mus musculus domesticus*

**DOI:** 10.1111/1365-2435.13354

**Published:** 2019-05-22

**Authors:** Christopher H. Taylor, Stuart Young, Jonathan Fenn, Angela L. Lamb, Ann E. Lowe, Benoit Poulin, Andrew D. C. MacColl, Janette E. Bradley

**Affiliations:** ^1^ School of Life Sciences University of Nottingham Nottingham UK; ^2^ IUCN SSC Asian Wild Cattle Specialist Group Chester UK; ^3^ Environmental Science Centre British Geological Survey Keyworth UK

**Keywords:** Diet, eco‐immunology, house mouse, immune response, stable isotope analysis

## Abstract

1. The ability, propensity and need to mount an immune response vary both among individuals and within a single individual over time.
2. A wide array of parameters has been found to influence immune state in carefully controlled experiments, but we understand much less about which of these parameters are important in determining immune state in wild populations.
3. Diet can influence immune responses, for example when nutrient availability is limited. We therefore predict that natural dietary variation will play a role in modulating immune state, but this has never been tested.
4. We measured carbon and nitrogen stable isotope ratios in an island population of house mice *Mus musculus domesticus* as an indication of dietary variation, and the expression of a range of immune‐related genes to represent immune state.
5. After accounting for potential confounding influences such as age, sex and helminth load, we found a significant association between carbon isotope ratio and levels of immune activity in the mesenteric lymph nodes, particularly in relation to the inflammatory response.
6. This association demonstrates the important interplay between diet and an animal's response to immune challenges, and therefore potentially its susceptibility to disease.

A plain language summary is available for this article.

## 1 INTRODUCTION

At any given time, an animal's “immune state” can be considered as the numbers, concentrations and distribution of the various cells and molecules that make up the immune system (Abolins et al., 2018). This immune state is highly variable both within and among individuals and is ultimately determined by the many immune challenges (i.e. antigens) encountered throughout the animal's life, as well as the individual's ability (Watson et al., 2016) and propensity (Jackson et al., 2014) to respond to them. While we have a good mechanistic understanding of how, for example, certain genes or nutrients might influence the immune response, less is understood about the relative importance of such influences in determining variation in the wild (Pedersen & Babayan, 2011). Elucidating the key drivers of this variation will play an important role in understanding susceptibility to many forms of disease.

We know through laboratory studies and food supplementation experiments that an animal's diet can have a major influence on its immune state. Immune responses are energetically costly (Lochmiller & Deerenberg, 2003), and therefore, the amount of energy acquired through the diet can influence the amount that is allocated to the immune system (Forbes et al., 2016). Furthermore, certain diet‐derived nutrients play a particularly important role in the immune system, so the quantities consumed can be a limiting factor in the strength of an immune response (Saino, Ferrari, Romano, Martinelli, & Møller, 2003; Webb, Leslie, Lochmiller, & Masters, 2003). Diet may also influence the gut microbiota, with far‐reaching consequences for the host's immune system (Murphy, Velazquez, & Herbert, 2015; Rosshart et al., 2017).

While experimental manipulations demonstrate that diet can influence immune state, there is a lack of evidence (outside of humans; see Barbaresko, Koch, Schulze, & Nöthlings, 2013) for whether natural dietary variation does indeed explain a significant proportion of variation in immune state. A recent study on wild house mice *Mus musculus domesticus* by Abolins et al. (2018) took important steps towards identifying the relative contributions of a range of host and environmental variables to immune state. Their analysis suggests that intrinsic host factors such as age and condition are more important than parasitic infections in influencing a wild animal's immune state. In addition, distinct populations show particular immune phenotypes, in a way which is not directly related to the extent of genetic differentiation. Importantly though, the study by Abolins et al. (2018) does not include any measures of the diet of the mice in question. Mice show a highly flexible diet (Sage, 1981), and we predict an association between natural dietary variation and immune state, but this remains to be tested.

Stable isotope analysis (SIA) is a method by which the proportions of stable isotopes of elements (such as carbon ^13^C:^12^C and nitrogen ^15^N:^14^N) can be used to determine certain key ecological parameters, including dietary variation (Ben‐David & Flaherty, 2012; Kelly, 2000; Peterson & Fry, 1987). Food sources vary in their isotope ratios, and this variation is incorporated by consumers in a predictable manner (DeNiro & Epstein, 1978, 1981). For example, differences in carbon isotope ratios can identify whether the diet is derived from a marine or terrestrial source (Hobson, 1987; Peterson & Fry, 1987), and differences in nitrogen isotope ratios can be used to determine dietary source and relative trophic position (Minagawa & Wada, 1984; Schoeninger, DeNiro, & Tauber, 1983). Importantly, SIA is a less biased means of estimating diet compared to other approaches such as gut content or faecal analysis, which are strongly influenced by the digestibility of the food items (Stapp, 2002). In addition, depending on the type of tissue sampled, SIA provides an estimate of the average diet over a period of weeks or months rather than a single snapshot in time (Tieszen, Boutton, Tesdahl, & Slade, 1983). On the other hand, a limitation of SIA is that while it provides an index of dietary variation, it does not identify the particular food sources involved unless a range of potential sources is also sampled in depth (see Methods: Stable Isotope Analysis for further details).

We aimed to investigate whether dietary variation in the wild house mouse *M. musculus domesticus* is associated with changes in immunological state. We measured carbon and nitrogen isotope ratios from mouse muscle tissue at a number of different geographical locations within a single island population, as a proxy for dietary variation. We characterized immune state by measuring expression of a number of immune‐related genes in the spleen and mesenteric lymph nodes (MLNs) and the concentration of various cytokines within the blood. We also recorded some habitat indices, mouse biometrics and gut parasite burdens. We predicted that isotope values will vary by sampling site, but that in addition they will be associated with changes in the immune state.

## 2 MATERIALS AND METHODS

### 2.1 Sample collection

**Figure 1:**
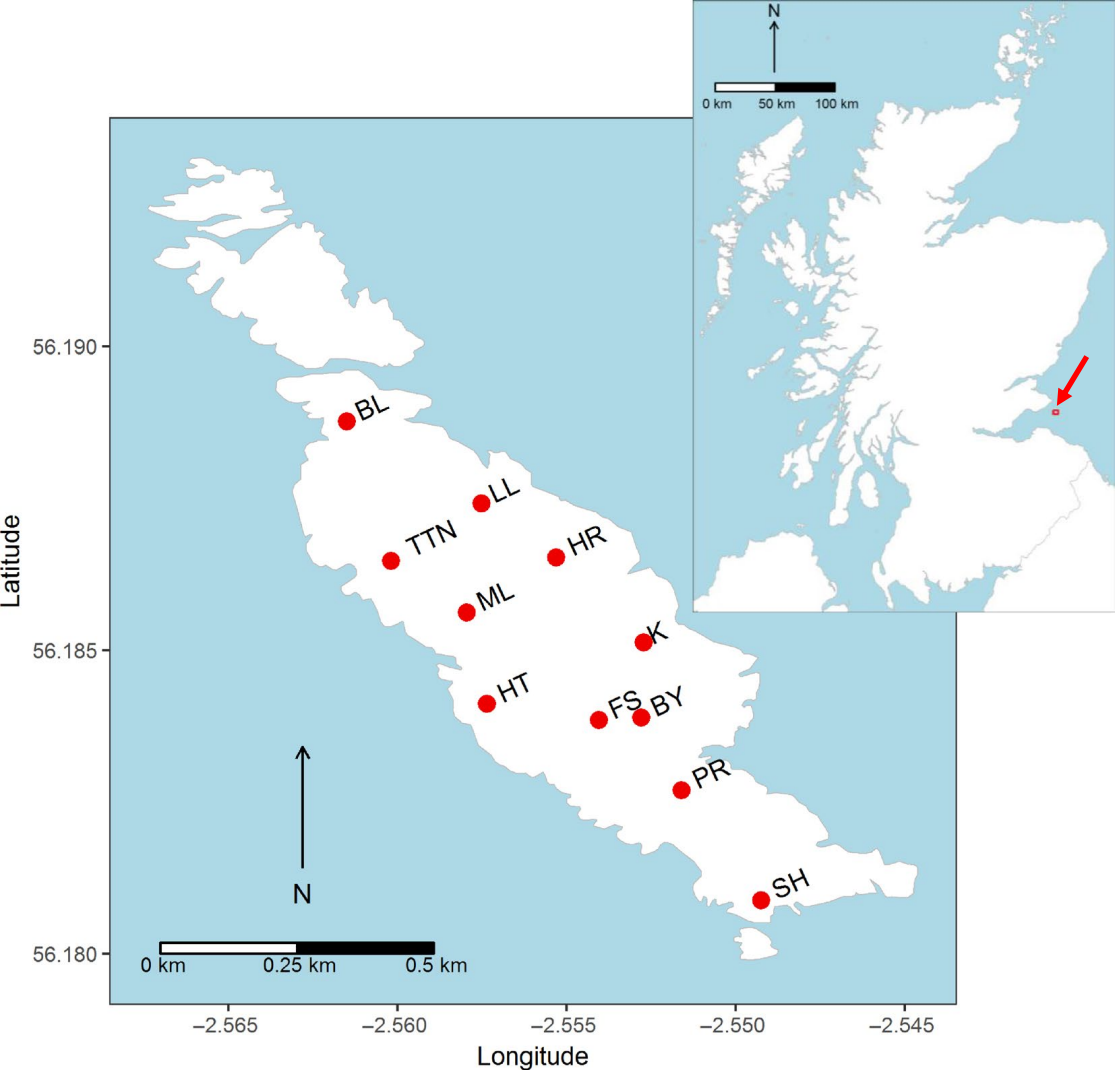
Approximate trapping locations on the Isle of May, along with the location of the isle within Scotland (inset). BL: Burnett's Leap; BY: Byres; FS: Fluke Street; HR: Holyman's Road; HT: High Tarn; K: Kettle; LL: Low Light; ML: Main Light; PR: Priory ruins; RO: Rona; SH: South Horn; TTN: Three Tarn Nick

We conducted fieldwork on the Isle of May (56°11'N, 2°33'W), an island off the coast of Scotland, UK, covering an area of 45 ha (Figure 1). The island is largely treeless and is mostly covered by different types of bird‐modified maritime grassland (Wright, Wal, Wanless, & Bardgett, 2010). Wild house mice are present on all parts of the island and are feral and non‐commensal (Triggs, 1991).

We trapped house mice over the course of 4 days between 9 and 12 October 2015. At each of eleven locations on the island (Figure 1), we placed 16 traps in pairs approximately every 2 m along a transect. We used primarily Longworth traps (Longworth Scientific Instrument Co.), along with small numbers of Ugglan (Granhab) and home‐made “Jordan” traps (Perrow & Jowitt, 1995). Equal proportions of trap types were used at each of the trapping locations in case there were any differences in trapping efficiency. All traps were baited with a commercially available wild bird seed mix, and hay was provided as insulation.

We checked the traps twice daily, and any non‐pregnant mice captured were taken to be culled. Mice were euthanized by a rising concentration of CO_2_ with death confirmed by exsanguination.

### 2.2 Habitat variables

To avoid over‐fitting models with large numbers of explanatory variables, we chose two key variables to represent habitat variation among sampling sites. Firstly, we categorized habitat type according to the dominant plant species found at each site, as this influences not only the plant food sources available but also other sources such as invertebrates. These categories were based on a vegetation survey carried out in 2008 (R. van der Wal, unpublished data); no major vegetation changes since that date were evident. Each site was dominated either by the perennial grass Yorkshire fog *Holcus lanatus* or by sea campion *Silene uniflora*.

We also recorded the density of breeding puffins, since, in other island populations, mice have been recorded scavenging and preying on seabird eggs and juveniles (Angel, Wanless, & Cooper, 2009; Cuthbert & Hilton, 2004). Furthermore, seabirds represent a potential source of marine‐derived nutrients in the diet of the mice, and such nutrients have a distinctive isotopic signature (Hobson, 1987). Values for “puffin density” are based on counts of occupied Atlantic puffin *Fratercula arctica* burrows from a census carried out in April/May 2017 (Newell, Harris, Burthe, & Daunt, 2017). There was no significant change in the Isle of May puffin population between 2013 and 2017 (Newell et al., 2017), so the distribution of occupied burrows here is likely representative of our study period. Census counts were divided among 27 different areas of the island, and we standardized each count by dividing by the area of the relevant region in hectares.

### 2.3 Mouse life history and physiology

We recorded sex, total body mass and snout–vent length (SVL) of each individual immediately after death. The eyes were removed and fixed in 10% formalin. We later dissected the eyes to remove the lenses which we dried at 60°C for 48 hr or until they showed no further weight loss (Rowe, Bradfield, Quy, & Swinney, 1985). The dry mass of each pair of lenses was used to estimate mouse age in days using the method from Rowe et al. (1985).

As a measure of condition, we calculated the Scaled Mass Index (SMI) from the body mass and SVL for each individual using the method described by Peig and Green (2009). SMI represents the equivalent value for body mass after allometric scaling to a standard body length and therefore represents excess or shortfall of mass for a given size. SMI correlates positively with the size of nutritional reserves (Peig & Green, 2009).

Circulating leptin levels were used as a further biomarker of physiological status and body condition (Abolins et al., 2018; Abolins, Pocock, Hafalla, Riley, & Viney, 2011). Serum samples were processed in duplicate with a custom Bio‐Rad Bio‐Plex mouse cytokine reagent kit according to manufacturer's protocol (Bio‐Rad). Along with detection antibodies for leptin, we also included detection antibodies for a number of cytokines in the multiplex assay (see “Immune markers” below).

Following incubation, the reaction mixture was analysed using a Bio‐Plex 200 Luminex‐based multiplex analysis system (Bio‐Rad). Unknown cytokine concentrations were calculated by Bio‐Plex Manager Software using standard curves derived from recombinant cytokine standards. Data that were below the assay's range of detection were assigned values of 0.001 (Abolins et al., 2017).

### 2.4 Parasite counts

The digestive tract was removed from each culled animal and stored individually in 70% ethanol. It was later dissected, and both gut contents and mucosa were examined for gastrointestinal helminths under a dissecting microscope. Helminths were identified to species level based on morphology. Two species were found in our samples: the pinworm *Syphacia obvelata* and the whipworm *Trichuris muris*. Juvenile and adult life stages of both sexes were all recorded and pooled as a single count.

### 2.5 Immune markers

We carried out qPCR to measure normalized mRNA expression of a suite of genes reflecting different functional arms of the immune system in spleen (14 genes) and MLN tissue (11 genes; for details, see Table S1). The spleen and MLNs were removed immediately following death and placed in RNAlater solution (Life Technologies). Samples were kept at 4°C for 24 hr; then, the supernatant was removed, and samples were stored at −80°C until extraction.

RNA was extracted from up to 30 mg spleen and MLN tissue using the NucleoSpin RNA kit (Macherey‐Nagel) following the manufacturers' protocol. The purity, concentration and integrity of RNA were assessed following Robertson, Bradley, and MacColl (2016). Synthesis of cDNA was performed on up to 2 µg of total RNA using the nanoScript2 Reverse Transcription Kit (Primerdesign), using a combination of oligo‐dT and random nonamer primers, following the manufacturers' protocol. All cDNA samples were diluted with nuclease‐free water (1:10 for spleen samples and 1:5 for MLN) and stored at −20°C before further use.

qPCRs were performed as described in Robertson et al. (2016), with primers designed and validated by Primerdesign (Southampton, UK). All samples were run in duplicate, with each plate also containing negative controls and a pooled reference sample. Due to technical issues, expression levels of some genes are only available from one tissue type (Table S1).

Six candidate endogenous “housekeeping” genes (*Actb*, *Gapdh*, *Rn18s*, *Rpl13a*, *Sdha* and *Ubc*) were assessed for stability by a geNorm assay (SYBR Green Kit; Primerdesign) against 15 randomly selected spleen or MLN cDNA samples. *Sdha* and *Ubc* were selected as the most stably expressed of the reference genes. The expression of the immunological target genes was normalized against the reference cDNA using the 2^−ΔΔ^ *^C^* ^T^ method (Pfaffl, 2001; Vandesompele et al., 2002).

In addition to the mRNA measurements, we measured circulating serum concentrations of nine cytokine molecules (IFN‐γ, IL‐1β, IL‐5, IL‐6, IL‐10, IL‐12β, IL‐13, IL‐17 and TNF‐α; see Table S1 for functions) using multiplex bead assay, following the method described for leptin under “Mouse life history and physiology” above.

### 2.6 Stable isotope analysis

Leg muscle tissue was taken from euthanized mice for use in SIA. Muscle tissue was used because it accurately reflects the isotopic composition of an animal's diet over several weeks or months, while tissues with a higher metabolic rate (e.g. liver) will reflect a shorter dietary period (Hobson & Clark, 1992; Kurle & Worthy, 2002; Tieszen et al., 1983). In the case of mice, the half‐life for carbon and nitrogen isotopes in muscle tissue is approximately 3–4 weeks (MacAvoy, Macko, & Arneson, 2005). This integration period ensured that our isotopic data were not too sensitive to noise caused by short‐term dietary variation, but reflected an “average” diet consumed over recent weeks.

All samples were kept frozen prior to drying and were then freeze‐dried at −50°C for approximately 12 hr before being ground to a fine powder with a mortar and pestle. Lipids were removed by soaking in a 2:1 chloroform:methanol solution (Cherry, Derocher, Hobson, Stirling, & Thiemann, 2011; Folch, Lees, & Sloane Stanley, 1957). Lipids typically have less ^13^C (DeNiro & Epstein, 1977), so lipid extraction reduces the risk of significant bias in δ^13^C values (Post et al., 2007; Tieszen et al., 1983).

Approximately 0.6 mg of prepared tissue from each sample was used in SIA. The isotope ratio mass spectrometry took place at the NERC Isotope Geosciences Facility (British Geological Survey, UK), measured on a continuous flow‐elemental analyser (Flash/EA) coupled to a Thermo Finnigan Delta Plus XL via a ConFlo III interface (all from Thermo Scientific). Isotope results were expressed as delta (δ) values, reported in per mil (‰) relative to international standards for δ^13^C (Vienna Pee Dee Belemnite (VPDB)) and δ^15^N (atmospheric nitrogen (AIR)), according to the following equation:

$$\delta X=\;\left[\left(\text{R}\_\text{sample}-\text{R}\_\text{standard}\right)-1\right]\times 1,000$$

where *X* is either ^13^C or ^15^N, and R_sample and R_standard are the ^13^C:^12^C or ^15^N:^14^N ratios of the sample or standard, respectively. δ^13^C and δ^15^N ratios were calibrated using an in‐house reference material M1360p (powdered gelatine from British Drug Houses, Poole, UK) with expected delta values of –20.32‰ (calibrated against CH7, IAEA) and + 8.12‰ (calibrated against N‐1 and N‐2, IAEA) for C and N, respectively. δ^13^C and δ^15^N analyses were undertaken in duplicate, and the average standard deviation of these pairs was δ^15^N = ±0.11‰ and δ^13^C = ±0.22‰. The 1σ reproducibility for mass spectrometry controls for these analyses was better than ±0.2‰ for both isotopes.

In our analysis, we used δ^13^C and δ^15^N values as proxies for dietary variation, without inferring specific details about the identity of the dietary sources. With sufficient data on the isotope values of potential food sources, it is possible to estimate the proportions consumed by each consumer (Parnell, Inger, Bearhop, & Jackson, 2010; Phillips, 2001). However, we are aware that δ^15^N values in island vegetation, and consequently further up the food chain, can be strongly influenced by sampling location over a fine spatial scale, at least in part due to the input of nitrogen from seabird guano (Cocks, Balfour, & Stock, 1998; Drever, Blight, Hobson, & Bertram, 2000; Mizutani & Wada, 1988; Wainright, Haney, Kerr, Golovkin, & Flint, 1998). We were therefore unable to infer the composition of an individual mouse's diet with any degree of certainty without taking large numbers of samples of each potential source type from each trapping location around the island. While such detailed sampling would undoubtedly have been informative, it was unfortunately not within the scope of the present study.

Instead, we have accounted for geographical variation in our models where necessary, such that remaining variation in δ^15^N and δ^13^C values is not attributable to the trapping location. We therefore made the reasonable assumption that the majority of the remaining variation corresponded to mice that are using food sources in different proportions, without identifying specifically what those sources were.

### 2.7 Statistical analysis

We analysed the relationship between ecological variables, including diet, and immune markers using redundancy analyses (Legendre & Legendre, 1998). Immune variables were divided into three groups: expression of immune genes in the spleen, expression of immune genes in the MLNs and cytokine protein concentrations measured from the blood. Each group was used as the set of response variables for a separate redundancy analysis. The immune variables were (log + 1) transformed to bring them close to a normal distribution, which ensured that the construction of ordination axes was not excessively influenced by extreme data points (Legendre & Legendre, 1998). Each redundancy analysis used the same set of predictor variables, all loosely related to the individual's ecology: δ^13^C, δ^15^N, age, sex, leptin concentration and presence/absence of *S. obvelata* and *T. muris*. Tests of significance for these ecological predictor variables were carried out against 9999 random permutations of the data and therefore did not depend on parametric assumptions (Legendre & Legendre, 1998).

To assess the relationship between diet and body condition, we conducted two linear mixed models with leptin concentration and SMI as the respective response variables, and δ^13^C and δ^15^N as predictors in each. Age, sex and presence/absence of *S. obvelata* and *T. muris* were also included as fixed effects and location as a random effect. We confirmed normality of residuals by inspection of a quantile–quantile plot and confirmed homoscedasticity on a scale‐location plot. We found no evidence for nonlinear relationships between continuous predictors (δ^13^C, δ^15^N and age) and response variables when examining plots of model residuals against predictor values. We also found no evidence for spatial correlation at the scale of sampling sites after plotting variograms of the model residuals against latitude and longitude coordinates (Legendre & Legendre, 1998). The contribution of the random effect was assessed via a likelihood ratio test by comparison with a linear model omitting the random term, and the random term was only retained if significant at *p* < 0.05. After this, we assessed all possible combinations of fixed effects using the R package MuMIn (Barton, 2016) and took the average of any resulting models for which ΔAIC (Akaike information criterion) < 2 as compared to the model with the lowest AIC value.

To examine associations between dietary variation and other ecological factors, we conducted two linear mixed models with δ^13^C and δ^15^N as the respective response variables. Fixed effects were age, sex, dominant vegetation, puffin density and presence/absence of *T. muris* and *S. obvelata*, with a random effect of sample site. Model checking, simplification and averaging were carried out as above.

### 2.8 Software

Statistical analysis was carried out in r version 3.4.4 (R Core Team, 2018), using packages tidyverse for data manipulation and visualization (Wickham, 2016), vegan for redundancy analysis (Oksanen et al. 2016), lme4 for linear mixed modelling (Bates, Mächler, Bolker, & Walker, 2015) and nlme for construction of variograms (Pinheiro et al. 2018).

## 3 RESULTS

### 3.1 Diet and immune response: low carbon isotope values are associated with stronger immune response in the MLNs

We conducted stable isotope analysis on 74 individual mice (21 females and 53 males; 24 juvenile and 50 mature). We found that variation in immune markers from the blood and the spleen was not explained by variation in ecological predictors (δ^13^C, δ^15^N, age, sex, leptin, *S. obvelata* and *T. muris*) (redundancy analysis; blood: *R* ^2^ = 0.13, Adjusted *R* ^2^ = 0.035, *p* = 0.16, *n* = 74; spleen: *R* ^2^ = 0.092, Adjusted *R* ^2^ = −0.024, *p* = 0.68, *n* = 63). However, we did find a significant association between MLN immune markers and our ecological predictors (*R* ^2^ = 0.20, Adjusted *R* ^2^ = 0.082, *p* = 0.019, *n* = 55). This association is driven by the δ^13^C values (pseudo‐*F* = 5.14, degrees of freedom = 1, 47, *p* = 0.0007); none of the other predictors contributed significantly.

**Figure 2:**
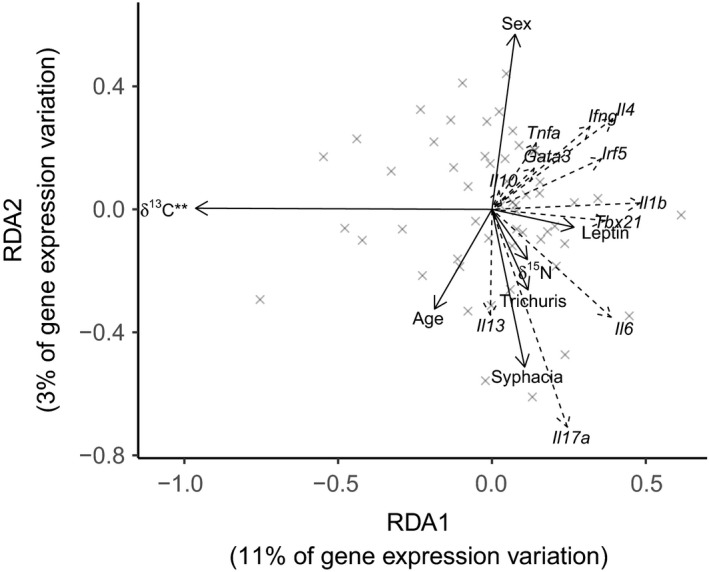
Triplot from a redundancy analysis of expression of immune‐related genes in the MLNs. Solid arrows indicate predictor variables, with **p < 0.001. Dashed arrows and italic labels indicate response variables. Grey crosses represent individual mice

To examine how δ^13^C value relates to gene expression in the MLNs in more detail, we examined the loadings of RDA1, which correlated very strongly with δ^13^C value (Figure 2, Table 1). RDA1 represented 11% of the measured variation in gene expression and 53% of the explained (as opposed to residual) variation and correlated positively with almost all of the genes included. In particular, five of the six most strongly correlated genes (*r* > 0.3) were linked to pro‐inflammatory or Th1 pathways (*Il1b*, *Il6*, *Tbx21*, *Irf5* and *Ifng*), and the three with the lowest correlation (*Gata3*, *Il10* and *Il13*) were linked to Th2 or anti‐inflammatory pathways. RDA1 also correlated strongly, but in the opposite direction, with δ^13^C. Therefore, mice with lower δ^13^C values tended to show higher levels of immune signalling in the MLNs, especially expression of genes with a connection to anti‐microbial inflammatory response.

**Table 1:** Correlations of predictor (ecological) and response (MLN gene expression) variables with RDA1 from a redundancy analysis. Variables are ordered by the magnitude of their correlation

Predictor	RDA1	Gene	RDA1
δ^13^C	−0.964	*Il1b*	0.482
Leptin	0.267	*Il4*	0.393
Age (days)	−0.187	*Il6*	0.387
*Trichuris* *muris* (present)	0.117	*Tbx21*	0.368
δ^15^N	0.115	*Irf5*	0.356
*Syphacia* *obvelata* (present)	0.106	*Ifng*	0.318
Sex (male)	0.075	*Il17a*	0.245
		*Tnfa*	0.144
		*Gata3*	0.137
		*Il10*	0.023
		*Il13*	−0.005

### 3.2 Diet and condition: low carbon isotope values are associated with higher levels of circulating leptin

**Figure 3:**
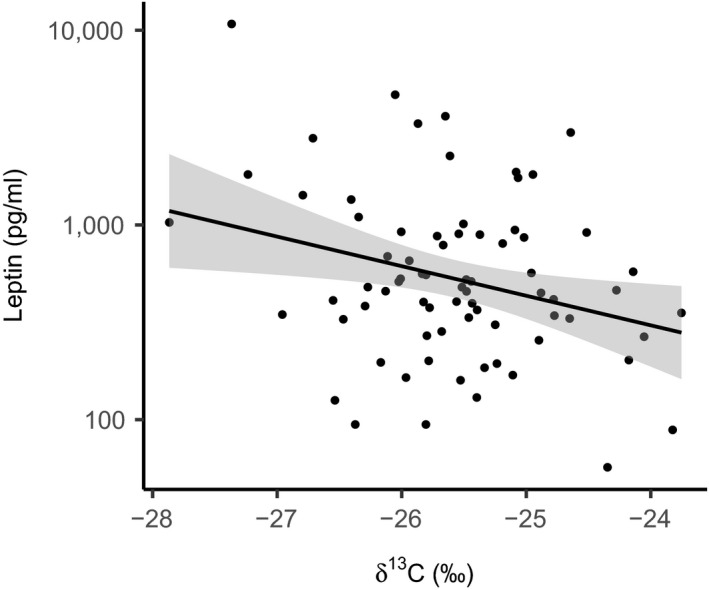
Variation in circulating leptin concentration with carbon isotope values. Points represent individual mice, and line shows model prediction with grey shading for ±*SE*

Although both leptin concentration in the blood and SMI can be considered indices of condition, they did not correlate with one another (Kendall's tau = 0.097, *p* = 0.22). We found a negative association between leptin concentration and δ^13^C value (coefficient (Coef) = −0.351, 95% confidence interval (CI) = −0.628 to −0.075; Figure 3 and Table 2), but no association with other predictors. We found no associations between SMI and isotope values, or other predictor variables (Table 2). The random effect of location was dropped from both the leptin and SMI models as it did not significantly improve fit (leptin ΔAIC = −0.82, *p* = 0.28; SMI: ΔAIC = 0.03, *p* = 0.15).

**Table 2:** Final coefficients for linear models of two condition variables (leptin concentration and Scaled Mass Index (SMI)) after model selection and averaging. Coefficients highlighted in bold are those for which the 95% confidence interval does not include zero

Term	Leptin				SMI			
	Coefficient	LCI	UCI	Weight	Coefficient	LCI	UCI	Weight
Intercept	−2.69	−9.78	4.40	NA	20.2	14.3	26.1	NA
δ^13^C	**−0.351**	**−0.628**	**−0.075**	1	−0.012	−0.218	0.194	0.086
δ^15^N					−0.034	−0.197	0.129	0.252
Sex					−0.114	−0.798	0.571	0.201
Age	0.0005	−0.0026	0.0037	0.185	0.0003	−0.0035	0.0041	0.091
*Syphacia* *obvelata*	0.018	−0.171	0.206	0.139	0.23	−0.65	1.10	0.345
*Trichuris* *muris*	−0.180	−0.676	0.315	0.5				

Note

LCI: lower 95% confidence interval; UCI: upper 95% confidence interval.

### 3.3 Nitrogen isotope values vary with location

**Figure 4:**
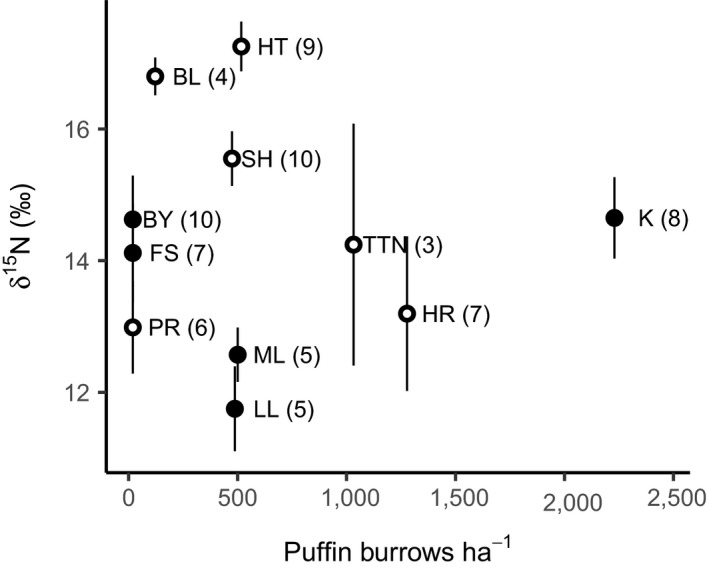
Variation in mouse nitrogen isotope values among trapping locations. Points show the mean value for each of the 11 locations, and vertical lines show ±*SE*. Letter codes refer to the sampling locations detailed in Figure 1, with sample size in brackets. Also shown are the density of occupied puffin burrows (*x*‐axis) and the dominant vegetation type (*Silene* *uniflora*, open circles; *Holcus* *lanatus*, filled circles). Of the variables shown here, only location explained a significant proportion of variation in δ^15^N values

The tested ecological variables did not explain any dietary variation in terms of carbon isotope values (Table 3). Trapping location was not retained in the δ^13^C model as it did not contribute to an improved fit (ΔAIC = −2, *p* = 1). In the case of δ^15^N, there was significant variation by trapping location (ΔAIC = 7.31, *p* = 0.0023; Figure 4, Table 3) but this was not associated with either puffin burrow density (Coef = 0.00001, CI = −0.00011 to 0.00014) or the dominant vegetation type (Coef for *S. uniflora* = −0.018, CI = −0.185 to 0.150). In addition, δ^15^N values were significantly higher in males (Coef = 1.14, CI = 0.19 to 2.09) and in individuals infected with *S. obvelata* (Coef = 1.03, CI = 0.04 to 2.01).

**Table 3:** Final coefficients for linear models of δ^13^C and δ^15^N after model selection and averaging. Coefficients highlighted in bold are those for which the 95% confidence interval does not include zero

Term	δ^13^C				δ^15^N			
	Coefficient	LCI	UCI	Weight	Coefficient	LCI	UCI	Weight
Intercept	−25.6	−25.8	−25.3	NA	12.4	10.7	14.1	NA
Sex	−0.014	−0.184	0.156	0.139	**1.140**	**0.190**	**2.090**	1.00
Age	0.00037	−0.00192	0.00265	0.194				0
Puffins	0.00001	−0.00011	0.00014	0.151				0
Vegetation (*Silene* *uniflora*)	−0.018	−0.185	0.150	0.151	1.10	−0.96	3.15	0.65
*Syphacia* *obvelata*				0	**1.03**	**0.04**	**2.01**	1.00
*Trichuris* *muris*				0				0

Note

LCI: lower 95% confidence interval; UCI: upper 95% confidence interval.

## 4 DISCUSSION

Here, we have shown that stable isotope values, very likely linked to natural dietary variation, are associated with the levels of stimulation for a wild mouse's immune system. Specifically, individuals with low values for δ^13^C show increased expression of immune‐related genes, particularly those associated with inflammatory responses. The effects appear to be local to the gut, as we observed these changes in the MLNs, but failed to find evidence for similar effects in the spleen or circulating blood. We found that the individuals with low δ^13^C also tended to have higher concentrations of leptin in the blood, although leptin alone did not explain the change in immune state. We found microgeographical variation in nitrogen but not carbon isotope values.

Our data support the prediction that diet is an important determinant of immune state. This adds to a recent body of work seeking to establish the sources of variation in immune state in wild animals (Abolins et al., 2018; Arriero et al., 2017; Pedersen & Babayan, 2011; Turner, Begon, Jackson, Bradley, & Paterson, 2011). We know that genetic variation underpins some variation in cytokine concentrations (Turner et al., 2011), but that ecological variables such as season and body condition also play a major role in determining immune state (Abolins et al., 2018). Given the known association between condition and immune state (Abolins et al., 2018) and evidence from food supplementation experiments (e.g. Forbes et al., 2016; Strandin, Babayan, & Forbes, 2018), it is unsurprising that dietary variation among individuals should be associated with the state of the immune system; nonetheless, to our knowledge our study is the first to show this under natural conditions in a non‐human animal (for humans, see, e.g., Barbaresko et al., 2013).

We note that, beyond the significant association described above, a large proportion (approximately 80% for the MLN data) of the variation in immune parameters remains unexplained in our model. Inevitably, in a wild observational study such as this one, there are myriad possible variables that might influence immune state, only some of which we can account for. Furthermore, cytokine concentrations can change over the course of days or even hours (Scheiermann, Kunisaki, & Frenette, 2013), whereas our isotopic data are reflective of dietary variation over the course of several weeks (MacAvoy et al., 2005). Despite this difference in time‐scale, we still observe an association, which suggests that changes to the immune state in this context may be somewhat persistent rather than acute responses to isolated antigens. An interesting future extension would be to examine the acute response in these wild mice directly, by challenging mice with deliberate and controlled introduction of antigens. Further immunological measures, such as functional blood cell counts, might also be informative as to the detailed nature of the immune changes observed.

Similarly to stable isotope studies in other species (e.g. Graves et al., 2012; Robertson, McDonald, Delahay, Kelly, & Bearhop, 2015; Mangipane et al., 2018), we found an association between isotope values and nutritional status, measured in our case by concentration of circulating leptin. However, it is often observed that different biomarkers of nutritional status yield conflicting results (Graves et al., 2012; Mangipane et al., 2018). While we found circulating leptin concentration correlated negatively with δ^13^C, the same was not true for SMI. The potential for inconsistency among biomarkers of nutritional status is well recognized, as different indices can reflect subtly different aspects of an animal's condition (Labocha, Schutz, & Hayes, 2014). Of the two measures used in our study, SMI may primarily reflect variation in mass of protein and water (Schulte‐Hostedde, Millar, & Hickling, 2001) while leptin is expected to correlate more closely with fat content (Frederich et al., 1995).

A connection between diet and inflammation has been well studied in mice in the laboratory due to associations with important aspects of human health such as obesity and diabetes (Murphy et al., 2015). The gut microbiota plays a pivotal role in low‐level gut inflammation (Cani et al., 2008), with high‐fat diets causing an increase in the proportion of bacteria of the phylum Firmicutes and stimulation of Toll‐like receptor 4, triggering inflammatory pathways (Kim, Gu, Lee, Joh, & Kim, 2012). We know that dietary variation also influences the gut microbial community in wild mice (Wang et al., 2014), and therefore, it is possible that the microbiota plays a role in mediating our observed association between diet and inflammation.

In theory, it is also possible that other gut organisms could provide a link between diet and immune response. For example, helminths (including *S. obvelata* and *T. muris* present in this study) can be acquired through feeding (Baker, 2007) and elicit a characteristic immune response from the host (Pritchard, Hewitt, & Moqbel, 1997), but in the case of this study we did not find any evidence for a difference in immune state between infected and uninfected individuals.

From our observational data on diet, condition and immune state, it is not possible to draw firm conclusions regarding causal relationships, as there are several different possible scenarios that are compatible with our data. For example, diet could affect immune state via changes in the individual's nutritional status (Forbes et al., 2016), or it could be that diet affects both immune state and nutritional status, but via largely independent mechanisms. It is even possible that immune state might be the cause of changes in feeding behaviour (Kyriazakis, Tolkamp, & Hutchings, 1998). In our opinion, given that the observed association appears to be specific to the MLNs rather than system‐wide, the most likely explanation is that dietary intake has separate effects on both immune state in the gut and nutritional status. Experimental evidence will be required to separate these hypotheses.

Unfortunately, a limitation of the present study is that we were not able to collect sufficient isotope data from potential food sources to characterize dietary composition: although we can use isotope values as a proxy for dietary variation, we can only hypothesize as to the food items involved. Variation in δ^13^C values is typically associated with differences in the photosynthetic origin of carbon in the food chain, for example discriminating between C3 and C4 producers (Ben‐David & Flaherty, 2012; Peterson & Fry, 1987). While there are no C4‐using plant species in this study system (*Holcus lanatus* is a C3 grass), in coastal or island locations such as here, low values of δ^13^C may indicate terrestrial producers and high values marine producers (Hobson, 1987). Therefore, the mice with high δ^13^C values that show poorer condition and lower levels of immune activity may have an unusually high proportion of “marine” food sources in their diet. The most likely candidate in this case would be seabird material: mice on the Isle of May have been observed scavenging on seabird carcasses (D. Steel and M. Newell, pers. comm. 2015), and both direct predation and scavenging of seabirds by mice have been recorded on subantarctic islands (Angel et al., 2009; Cuthbert & Hilton, 2004). We speculate that these marine food sources may be of poorer quality than terrestrial sources such as plants or invertebrates, leading to the poorer condition of the individual mice that consume them. Alternatively, mice already in poor condition might be subject to intraspecific competition forcing them to switch to consuming the marine food sources, as observed in another island population of *M. musculus* (Cuthbert et al., 2016). Further study is required to determine the dietary sources in more detail.

It is worth emphasizing that our observations of immune state were not consistent among tissues, in that the effects observed in the MLNs were not reflected in the spleen or blood data. We must therefore consider that any outcomes for the individual in terms of altered disease susceptibility are likely also to be primarily local to the gut region.

## AUTHORS' CONTRIBUTIONS

S.Y., J.F., A.D.C.M. and J.E.B. conceived the ideas and designed methodology; S.Y., J.F., A.E.L. and B.P. carried out fieldwork and immunological assays; A.L.L. carried out stable isotope analysis; C.H.T. and S.Y. conducted statistical analysis of the data; C.H.T. led the writing of the manuscript; and C.H.T. and S.Y. contributed equally to this publication and should be considered joint first authors. All authors contributed critically to the drafts and gave final approval for publication.

## DATA ACCESSIBILITY

Data deposited in the Dryad Digital Repository: https://doi.org/10.5061/dryad.3ng4kr8 (Taylor et al. 2019).
