# Supplementary material for: Immune state is associated with natural dietary variation in wild mice Mus musculus domesticus
Source: Funct Ecol. 2019 May 22;33(8):1425–35. doi: 10.1111/1365-2435.13354 (PMC6767599; doi:10.1111/1365-2435.13354)
Supplement: Supplementary file 1 [file FEC-33-1425-s001.pdf]

Functional Ecology

# A wild mouse's immune state is linked to its diet

*Christopher H. Taylor, Stuart Young, Jonathan Fenn, Angela L. Lamb, Ann E. Lowe, Benoit Poulin, Andrew D. C. MacColl and Janette E. Bradley*

An animal's immune system is made up of a complex array of cells and molecules that enable them to respond to foreign material (such as harmful micro-organisms) in their body. At any given time, the numbers of these cells and molecules determine an individual's "immune state", which is closely linked to health. We know from carefully controlled laboratory experiments that a number of different factors, such as diet, gut microbes and age, can under certain circumstances influence an animal's immune state. But less is known about the importance of these different factors in wild populations, where levels of variation may be different and interactions may be more complex.

We set out to investigate whether wild mice that naturally feed on different food sources have different immune states. We captured mice on the Isle of May in Scotland and measured a range of immune signalling molecules in their blood, spleen and lymph nodes next to the gut. As food sources vary in their composition of carbon and nitrogen isotopes, we were able to estimate the past diet of the mice by looking at isotope proportions that had been integrated into their muscles. We found that various immune molecules close to the gut, particularly those that can trigger inflammation, were associated with diet. It is becoming clear, through this and other recent studies, that wild animals' immune state does not just depend on major events such as infections but also on everyday factors such as diet.

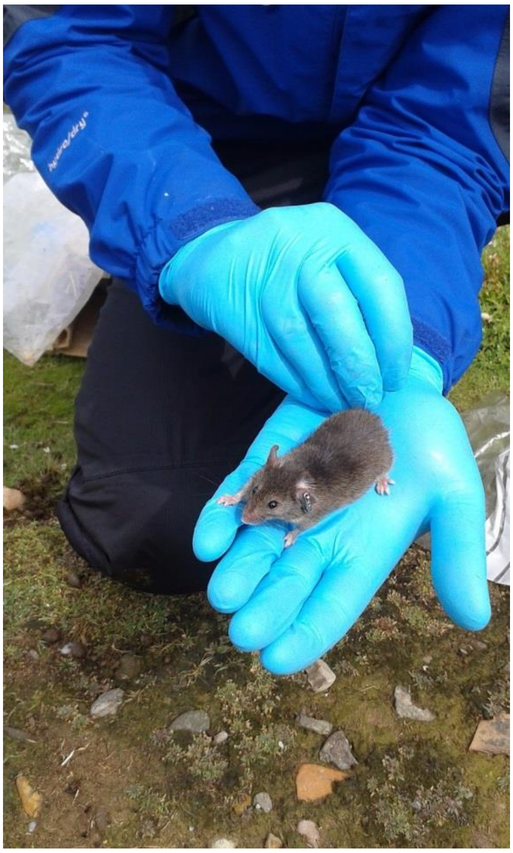

*Photo credit: Jonathan Fenn. "A large male house mouse trapped on the Isle of May, Scotland."*
